# Phytochemical Profile and Biological Effects of Spruce (*Picea abies*) Bark Subjected to Ultrasound Assisted and Microwave-Assisted Extractions

**DOI:** 10.3390/plants10050870

**Published:** 2021-04-26

**Authors:** Adrian Nisca, Ruxandra Ștefănescu, Diana Ionela Stegăruș, Anca Delia Mare, Lenard Farczadi, Corneliu Tanase

**Affiliations:** 1 Doctoral School of Medicine and Pharmacy, “George Emil Palade” University of Medicine, Pharmacy, Sciences and Technology of Târgu Mures, 38 Gheorghe Marinescu Street, 540139 Târgu Mureș, Romania; 2 Department of Pharmacognosy and Phytotherapy, “George Emil Palade” University of Medicine, Pharmacy, Sciences and Technology of Târgu Mures, 38 Gheorghe Marinescu Street, 540139 Târgu Mureș, Romania; 3 National Research and Development Institute for Cryogenics and Isotopic Technologies—ICSI Râmnicu Vâlcea, 4th Uzinei Street, 240050 Râmnicu Vâlcea, Romania; diana.stegarus@icsi.ro; 4 Department of Microbiology, “George Emil Palade” University of Medicine, Pharmacy, Sciences and Technology of Târgu Mures, 38 Gheorghe Marinescu Street, 540139 Târgu Mureș, Mureș, Romania; anca.mare@umfst.ro; 5 Chromatography and Mass Spectrometry Laboratory, Center for Advanced Medical and Pharmaceutical Research, “George Emil Palade” University of Medicine, Pharmacy, Sciences and Technology of Târgu Mures, 38 Gheorghe Marinescu Street, 540139 Târgu Mureș, Romania; lenard.farczadi@umfst.ro; 6 Department of Pharmaceutical Botany, “George Emil Palade” University of Medicine, Pharmacy, Sciences and Technology of Târgu Mures, 38 Gheorghe Marinescu Street, 540139 Târgu Mureș, Romania; corneliu.tanase@umfst.ro

**Keywords:** *Picea abies*, phenolic compounds, antioxidant, terpenoids, catechin, antibacterial activity

## Abstract

The bark of various tree species is a byproduct of the forestry industry that is not used at its full potential, considering the wide range of phytochemicals that are contained in these vegetal matrices and the health benefits that these compounds could provide for society. Our goal was to assess and compare the phytochemical composition of some hydroalcoholic spruce (*Picea abies*) bark extracts attained by ultrasound assisted extraction (UAE) and microwave-assisted extraction (MAE) and their antioxidant and antibacterial effects. The levels of total phenolics and tannins in the bark extracts were determined using methods based on the Folin–Ciocâlteu reagent, while specific phenolic and volatile compounds were identified and quantified using an UPLC-PDA method and a GC-FID method, respectively. After the chemical composition assessment, the antioxidant capacity (AC) was evaluated by measuring the scavenging ability against two free radicals (DPPH and ABTS). The minimum inhibitory concentration (MIC) was determined to assess the antibacterial activity of the extracts. The results indicated that the extracts produced by UAE had higher contents of polyphenols and tannins and also a higher content of the main phenolic compounds identified, catechin and epicatechin, compared to the MAE extracts. In contrast the highest content of volatile terpenoids (mainly α- and β-pinene) was found in the MAE extracts. All of the tested extracts exhibited relatively high antioxidant activities (especially the UAE extracts) and low MICs against Gram-positive bacteria but were mildly efficient against Gram-negative bacteria. These findings show that the spruce bark might be an important source of bioactive compounds that can be easily extracted from these industrial secondary products. Various uses of this vegetal material may emerge, due to its antioxidant and antibacterial effects.

## 1. Introduction

For the entirety of human history, different tree barks were used for various purposes, including medicinal purposes such as the treatment or the prevention of many pathologies [1,2]. Despite the health benefits provided by these raw materials, most of the bark’s parts that result after wood processing are still inefficiently used for thermal energy [3,4]. Thus, a lot of recent research in this field has been focused on the evaluation of the phytochemical content and biological activities of the extracts attained from various tree species barks [2,5,6], with several studies indicating numerous classes of bioactive compounds, mainly polyphenols [4,7].

Polyphenols are secondary metabolites that are often found in the vegetal foodstuff [8], but lately, their presence was indicated in different tree barks [9,10]. They have an essential role in the protection of the plant against external threats, such as bacterial and fungal pathogens, herbivores, and insects [11,12].

Moreover, these secondary metabolites are not only essential for plant protection and environmental integration of the containing species, but also possess various biological activities that may benefit human health, such as antioxidant [13,14,15], antibacterial [14,16], antiviral [17,18,19], antifungal [20,21,22], and antitumor [9,10,23] effects.

One of the most important properties of phenolic compounds is their antioxidant capacity, provided by their phenolic hydroxyl groups that can donate electrons to neutralize free radicals. These reactive molecular species may cause DNA damage or lipids’ oxidation in humans [2,9], processes that are often linked to different pathologies such as diabetes, neurodegenerative disorders, cardiovascular and respiratory diseases, and cancers [24]. By neutralizing these free radicals, polyphenols are considered useful for the prophylaxis of these health problems [25]. Moreover, the antibacterial activity and synergistic effects of polyphenols along with synthetic antibiotics [24,25,26,27,28] may become useful in the management of various bacterial infections with multidrug-resistant bacterial strains that are emerging at a fast pace nowadays [29,30].

However, in order to use these phytochemicals, for their beneficial effects, they must be extracted from the vegetal matrices first. For a long time, traditional methods like maceration and Soxhlet extraction were used for extraction. Still, these methods proved to be time and energy consuming [31]. Nowadays, these conventional methods are enhanced using microwave radiations and ultrasounds to reduce time, energy, and solvent consumption [10]. The ultrasound assisted extraction (UAE) is commonly used as it does not require complex equipment and is simple to use, while efficiently solving the problems of the classic methods, but compared to other modern methods this one is the most time and solvent consuming [32]. This method is based on the cavitation process caused by the ultrasounds passing through the solvent. The process leads to the formation of cavitation bubbles near the surface of the vegetal matrix. When these bubbles collapse they damage the cellular walls’ structure, increasing the contact surface of the matrix and solvent by increasing the cellular wall permeability, thus enhancing the diffusion process and extraction yield [33,34]. The microwave assisted extraction (MAE) on the other hand uses the energy of the microwave radiation to heat the entire sample volume (solvent and matrix) at once. The fast raise of internal temperature in the vegetal matrix leads to an increased intracellular pressure which damages the cellular walls, releasing the internal cellular content, while the raise in solvent temperature accelerates the diffusion process [33]. This method has a high extraction and time efficiency and needs low solvent amounts, but can be used only for the extraction of low molecular weight, polar compounds that are stable at high temperatures [35].

*Picea abies* L. (Norway spruce) is a species of the Pinaceae family; previous studies identified in its bark various phenolic compounds such as gallic, vanillic, syringic, *p*-coumaric, ferulic, synapic acids, taxifolin, quercetin, and catechin [36,37], but also terpenoids (β-sitosterol, γ-sitosterol, thunbergol, α-pinene, β-pinene, and limonene) and stilbenes (piceid and astrignin) [38,39]. The presence of such compounds in spruce bark might explain the extracts’ antioxidant, antibacterial, antifungal and antitumoral (against human melanoma cells) activities [9,40,41].

Although, data about the cytotoxicity of *P. abies* bark extracts could not be found, a study that analyzed the *P. mariana* bark hot water extracts did not show any cytotoxic effects on RAW 264.7 macrophages and, even though high levels of phenolic compounds may exert pro-oxidant effects in vitro, due to the overproduction of H_2_O_2_, quinones, and semiquinones, which might lead to cellular death, these effects are unlikely to happen in vivo, because of the biotransformation of these compounds [42,43].

Considering the data presented in the literature, the bark of *P. abies* might be an important forestry waste material, containing several bioactive compounds that could have beneficial health implications, due to their biological effects.

Therefore, this study focused on the evaluation of the phytochemical profile and biological effects of the extracts attained by different extraction methods and from *P. abies* barks collected in different locations. A comparison between the two extraction methods and between the different collecting sites was performed. For the chemical composition assessment, the Folin–Ciocâlteu method was performed to measure the levels of polyphenols and tannins, while UPLC-PDA and GC-FID methods were performed to identify the main phenolic compounds and volatile terpenoids, respectively. As for the biological activity, the antioxidant capacity (AC) and antibacterial activity were assessed by the DPPH and ABTS assays and by the microdilution assay, respectively.

## 2. Results and Discussions

### 2.1. Assessment of the Content of Total Polyphenols (TPC)

**Figure 1:**
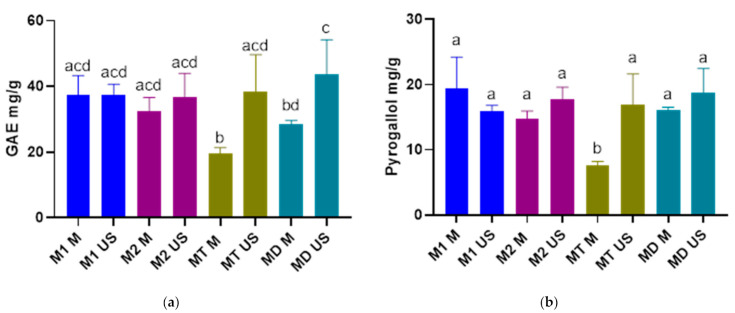
(**a**) Total polyphenol content (TPC) of spruce bark extracts; (**b**) total tannin content (TTC) of spruce bark extracts; significant differences are represented by different letters (significance level α = 0.05); M1 US—*P. abies* (Maramureșului Mountains National Park site 1) extract produced by UAE, M2 US—*P. abies* (Maramureșului Mountains National Park site 2) extract produced by UAE, MT US—*P. abies* (Tașca) extract produced by UAE, MD US—*P. abies* (Dolhasca) extract produced by UAE, M1 M—*P. abies* (Maramureșului Mountains National Park site 1) extract produced by MAE, M2 M—*P. abies* (Maramureșului Mountains National Park site 2) extract produced by MAE, MT M—*P. abies* (Tașca) extract produced by MAE and MD M—*P. abies* (Dolhasca) extract produced by MAE.

The results of the spectrophotometric determination of the spruce bark extracts TPC are presented in Figure 1a, as mg of GAE per g of vegetal material.

As it can be observed, the MD US variant had the highest TPC (43.64 ± 10.52 mg/g), but the content was significantly higher only when compared to MT M and MD M. The lowest TPC was recorded for the MT M variant (19.54 ± 1.79 mg/g), having significantly lower levels of phenolic compounds in comparison to the other variants excepting MD M. It can be also noted that the extracts attained via UAE are associated with higher TPC. Every variant obtained using this extraction method had higher TPC than the variants obtained by MAE, however the only significant differences occurred between the MT (US and M) and MD (US and M) experimental variants. Although slight differences occurred between the different collecting sites, there were no significant differences registered regarding the TPC.

Other studies regarding the TPC of spruce bark extracts attained by UAE achieved similar results. For example a study that used almost identical parameters for the UAE of the *P. abies* bark, identified a TPC of around 40 mg GAE/g, indicating the similarity of the results [44]. Another baseline study, that also focused on the UAE of the *P. abies* bark, achieved a TPC of the extracts of 28.73 ± 0.08 mg GAE/g, thus recording a lower level of phenolic compounds compared to most of our experimental variants. However, in this baseline study an aqueous extraction was performed and not a hydro-alcoholic one, using a higher extraction temperature (70 °C) and a lower extraction time (15 min) compared to our extraction parameters [45].

Sládková A. et al. conducted a study on *P. abies* bark extracts attained by MAE, indicating a maximum TPC of 321.1 mg GAE per 100 g dry bark. Although these results showed a lower yield of phenolic compounds compared to our results for the extracts obtained by MAE, it must be mentioned that a different MAE protocol was used [46].

These data show that the UAE might be a more efficient extraction technique to achieve higher yields of phenolic compounds from the bark of *P. abies*.

### 2.2. Assessment of the Content of Total Tannins (TTC)

The results regarding the TTC are represented in Figure 1b, as mg pyrogallol per g of vegetal material. It has been noticed that the highest TTC was recorded for the M1 M variant (19.34 ± 4.88 mg/g); however, no significant difference occurred between the experimental variants except MT M, for which a significantly lower level of tannins (7.56 ± 0.65 mg/g) was identified. Although almost no significant differences were registered after comparing the extracts, the results showed that slightly higher TTCs were associated with the extracts obtained by UAE, except the M1 variants, for which, the MAE seemed to lead to a slightly higher tannin yield, but the difference was not significant. As in the case of TPC, minor differences occurred between the collecting sites, but these differences were not significant.

Coșarcă S.L. et al. quantified the tannin content of their aqueous spruce bark extracts attained by UAE and achieved a concentration of 11.4 mg pyrogallol/g, thus indicating a lower level of TTC compared to our experimental variants [9]. Although the methods used in this baseline study were similar to the methods used in the present study, the difference in the extraction time, temperature and solvent might be the reason for the disparity of the results. Data about the tannin content of spruce bark extracts attained by MAE could not be found in the literature.

Other studies regarding the tannin content of spruce bark were found, but these studies focused on the aqueous extraction of the spruce bark at pilot scales [47,48].

### 2.3. Terpenoids Content

After the GC-FID analysis, the volatile compounds presented in Table 1 were identified and quantified, the results being expressed as µg compound per g vegetal material. It was observed that the main terpenoids identified in the spruce bark were α-pinene and β-pinene, the M1 M variant having the significantly highest α-pinene content (2198.33 ± 0.25 µg/g), while the MD M variant had the highest β-pinene content (4312.24 ± 4.07 µg/g). As for the less abundant identified compounds, the M2 M variant had the highest content of camphene, α-phellandrene, myrcene, and tricyclene, the MT M variant contained the highest yield of 3-carene and limonene, while the MD M variant also had the highest sabinene content out of all tested extracts. The most part of the results showed that higher yields of volatile compounds were associated with the variants obtained with the MAE method, however exceptions were identified too. For example, higher levels of camphene and myrcene were measured in the MT US variant compared to MT M and 3-carene content was higher in the UAE variants compared to the MAE counterparts, excepting the MT variants. Moreover, α-phellandrene and limonene contents were higher in the M1 US and M2 US, respectively, compared to their MAE counterparts.

In the case of volatile terpenoids content, the collecting site had a significant influence. For example, it can be seen in Table 1 that the spruce bark collected from Dolhasca (MD) had the significantly highest content of β-pinene, while the spruce bark collected from site 1 of Maramureșului Mountains National Park had the highest α- pinene content even when compared to the second site of the same area.

In a study conducted on spruce bark extracts attained by Soxhlet and super-critical CO_2_ extractions, the terpenoids were identified and quantified, but none of the compounds identified in this study corresponded to their results [49]. However, two other studies that focused on the emissions of volatile organic compounds from the spruce bark have indicated that high levels of α- and β-pinene were emitted from the spruce barks, but also lower quantities of limonene, 3-carene, camphene, sabinene, α-phellandrene, and tricyclene were identified [50,51], these results are also similar with our findings.

### 2.4. Individual Polyphenol Content

The major peaks occurring at retention times of 4.53 min and 5.25 min were catechin and epicatechin. The calculated contents of catechin and epicatechin are found in Table 2. For the UAE samples higher contents of catechin and epicatechin were identified, while for the MAE variants, a lower yield of these compounds was registered. Consistent with the results obtained in TPC and tannin content, M1 and MD samples contain the highest concentrations of both catechin and epicatechin, closely followed by MT samples. High catechin and epicatechin concentrations were expected, since they are the main monomers of spruce bark proanthocyanidins [47,48].

None of the samples had detectable levels of gallic, sinapic, and vanillic acids, neither taxifolin, although previous reports suggested that these compounds are among the polyphenols found in spruce bark extracts [44]. These polyphenolic compounds are, however, susceptible to degradation during sample preparation and extraction. Moreover, our results clearly suggest that the UAE is more efficient for the extraction of catechin and epicatechin from the spruce bark. In contrast Albuquerque et al., showed that the microwave assisted extraction is more efficient for the extraction of catechin compared to the ultrasound assisted extraction [52]. This discrepancy may appear, because a different vegetal matrix was used in the baseline study and also a more controlled environment was used for the MAE. However, the degradation of catechin and epicatechin in our MAE variants is unlikely due to the fact that these compounds suffer modifications only at higher temperatures than those achieved by us in the MAE [53].

### 2.5. Antioxidant Capacity (AC)

#### 2.5.1. DPPH Assay

**Figure 2:**
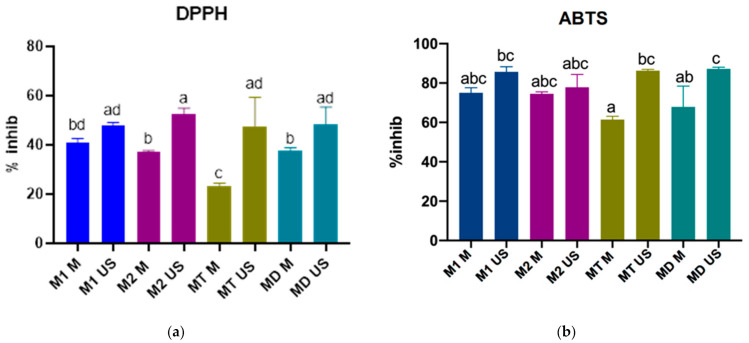
(**a**) Antioxidant capacity (AC) assessed by the DPPH method; (**b**) antioxidant capacity (AC) assessed by the ABTS method; significant differences are represented by different letters (significance level α = 0.05); M1 US—*P. abies* (Maramureșului mountains national park site 1) extract produced by UAE, M2 US—*P. abies* (Maramureșului Mountains National Park site 2) extract produced by UAE, MT US—*P. abies* (Tașca) extract produced by UAE, MD US—*P. abies* (Dolhasca) extract produced by UAE, M1 M—*P. abies* (Maramureșului Mountains National Park site 1) extract produced by MAE, M2 M—*P. abies* (Maramureșului Mountains National Park site 2) extract produced by MAE, MT M—*P. abies* (Tașca) extract produced by MAE and MD M—*P. abies* (Dolhasca) extract produced by MAE.

To assess the AC the DPPH method was used, and the results of the assay, expressed as inhibition percentages of the DPPH free radical, are represented in Figure 2a. These inhibition percentages correspond to 0.15 mg vegetal material and 0.1 mg DPPH reagent per tested sample.

**Figure 3:**
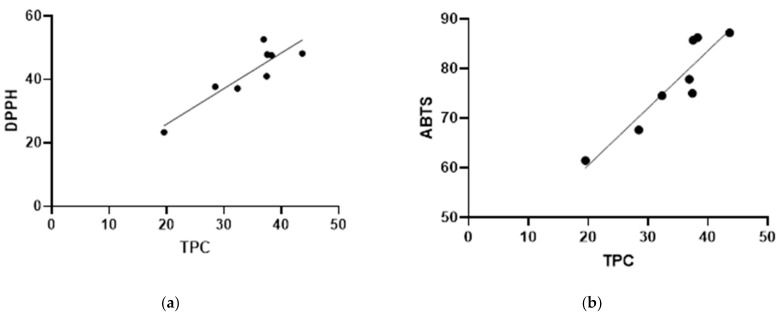
(**a**) Correlation between the TPC and inhibition percentages (DPPH assay) R^2^ = 0.8006; (**b**) Correlation between the TPC and inhibition percentages (ABTS assay) R^2^ = 0.8484.

It was shown that the extracts attained by UAE exhibit higher ACs compared to their MAE counterparts. The M2 US variant had the highest AC (52.64% ± 2.35%) between all extracts obtained by UAE, however the differences were not statistically significant. However, when compared to MAE variants, M2 US had a significantly higher AC. M1 M was the only MAE variant that had a comparable AC with the UAE variants, these results indicating a correlation between the AC and TPC. This correlation is further reinforced by the fact that MT US and MD US had significantly higher Acs than their MAE counterparts, but also significantly higher TPC values, suggesting that higher polyphenolic content may lead to a stronger antioxidant activity. The results of the Pearson test presented in Figure 3a have also confirmed that a correlation exists between the TPC and the inhibition percentages registered in the DPPH assay (R^2^ = 0.8006).

Previous studies have also indicated the DPPH radical scavenging ability of the spruce bark extracts attained by the UAE [9,37], but unfortunately no data were found describing this activity in spruce bark extracts obtained by MAE.

#### 2.5.2. ABTS Assay

The inhibition percentages registered for the ABTS assay indicate the AC corresponding to 1.25 mg spruce bark and 0.17 mg ABTS reagent per tested sample. These results are presented in Figure 2b, showing that the extracts obtained by UAE have a superior AC compared to their MAE counterparts. Although between M1 M and M1 US, and M2 M and M2 US no significant difference occurred by comparing their AC resulted after the ABTS assay, the differences between the MT M and MT US, and MD M and MD US are significantly different, indicating a similar trend as in the results of the previously performed antioxidant activity assay. Moreover, like in the previous assay, the variants that had higher polyphenol contents have led to a superior AC, the Pearson test indicating even a stronger correlation between the TPC and the inhibition percentages resulted in the ABTS assay (R^2^ = 0.8484), as shown in Figure 3b.

The correlations registered between the TPC and the Acs recorded for both assays indicate that polyphenols are mainly responsible for the antioxidant properties of the bark extracts, even though the bark is a complex matrix containing a mix of phytochemical compounds.

Regarding the differences between the AC of samples collected from different sites, slight variations were registered, however none of them were statistically significant.

A previous study also highlighted the AC of ethanolic spruce bark extracts obtained by UAE, tested with the ABTS reagent [54]; however, no studies measuring the AC of spruce bark extracts obtained by MAE with the ABTS assay, were found. For both assays (DPPH and ABTS), the results of this study and those of the baseline studies, could not be directly compared because of the different methodologies used and the different ways to express the final results.

As previously stated our results suggested a positive correlation between the TPC and AC of the tested extracts, however other studies that analyzed the relation between the two variables found no significant correlation [55,56,57]. It is necessary to mention that the two studies that analyzed this correlation on spruce bark extracts used deep eutectic solvents, while the third study focused on *Rosmarinus officinalis* ethanolic extracts. Moreover, studies that compared the TPC and AC of extracts attained by UAE and MAE showed that the MAE is more efficient for extracting polyphenols and results in extracts with superior AC [58,59,60]. In contrast, our findings showed that the UAE is more efficient regarding the TPC and AC. The discrepancy between our results and the data found in literature might be due to the different methodologies used for extraction, or even because the vegetal matrices used in the baseline studies were not barks. For example, in one of our previous works conducted on pine bark, it was also shown that the UAE is more efficient for extracting polyphenols and for achieving extracts with higher antioxidant capacities [61].

### 2.6. Antibacterial Activity

The antibacterial activity of the spruce bark extracts was assessed against Gram-positive and Gram-negative bacterial strains, by determining the MIC of the extracts, in order to evaluate the efficiency of each experimental variant against each tested strain. The results of the antibacterial activity are presented in Table 3.

#### 2.6.1. Gram-Positive Bacteria

As it can be observed M1 M and MD M were the most efficient variants against Gram-positive bacterial strains (*Staphylococcus aureus* and methicillin resistant *Staphylococcus aureus*-MRSA), having the lowest MIC (3.125 mg/mL) against both strains. The M2 US and MD US variants also had the lowest MIC value, but only against one of the two tested strains. The Gram-positive bacterial strains were inhibited by all tested variants, roughly with the same efficiency.

#### 2.6.2. Gram-Negative Bacteria

A modest antibacterial activity was identified against the tested Gram-negative bacterial strains (*Klebsiella pneumoniae*, *Pseudomonas aeruginosa*, and *Escherichia coli*). For the extracts attained by UAE all MICs were equal to, or above the concentration of the raw, undiluted extract (50 mg/mL). M1 US, MT US, and MD US exhibited antibacterial activity against *E. coli*, none of the UAE extracts were efficient against *K. pneumonia*, and all of them showed antibacterial properties against *P. aeruginosa* at the highest concentration (50 mg/mL). Similarly, the extracts obtained by MAE had MICs equal or above the maximum concentration tested (100 mg/mL) as it can be seen in Table 3.

In a previous study, it has been shown that aqueous spruce bark extracts obtained by classical extraction and UAE had antibacterial effects against Gram-positive bacteria, but these extracts were also efficient against *K. pneumoniae* and *P. aeruginosa* at an MIC of 15 mg/mL or lower [45]. Another study showed that spruce bark extracts obtained by Soxhlet and supercritical carbon dioxide extractions may inhibit the growth of *Enterococcus faecalis* and *Streptococcus thermophilus*, because of the content in catechin, taxifolin, astringin, and isorhapontin [62]. These results suggest that our spruce bark extracts may exhibit this antibacterial activity because of the catechins present in the natural extracts, as other studies have also shown the antibacterial properties of these compounds against bacterial strains such as *S. aureus* and *E. coli* [63,64].

## 3. Materials and Methods

### 3.1. Materials

The spruce barks used to attain the tested extracts, were gathered in the beginning of spring from several sites in Romania. The collecting sites were Tașca (46°53′34″ N, 26°02′05″ E), Neamț county, Dolhasca (47°25′49″ N, 26°36′34″ E), Suceava county and two sites in the area of Maramureșului Mountains National Park (47°47′05″ N, 24°34′20″ E), Maramureș County. The itinerary method was used to gather bark samples from randomly selected individuals from each site. The vegetal material was collected from a trunk height of approximately 1.5 m. A voucher specimen was deposited at the Herbarium of the Pharmaceutical Botany Department, George Emil Palade, University of Medicine, Pharmacy, Sciences and Technology of Tîrgu-Mureş, with reference number: 116/3–116/6. The final step of the plant material preparation consisted in the grinding of the spruce bark samples collected with a GRINDOMIX GM 200 mill (Retsch GmbH, Haan, Germany).

Gram-positive and Gram-negative bacterial strains were used to assess the antibacterial activity of the natural extracts. *Staphylococcus aureus* (ATCC 25923), methicillin-resistant *Staphylococcus aureus* (ATCC 43300), *Escherichia coli* (ATCC 25922), *Klebsiella pneumoniae* (ATCC 700603), and *Pseudomonas aeruginosa* (ATCC 27853) strains were used and provided by the Microbiology Department from the “G.E. Palade” University of Medicine, Pharmacy, Sciences and Technology from Târgu-Mureș.

Reagents like ethanol 96% (*v*/*v*) used to obtain 50% and 70% solutions utilized for extractions and Na_2_CO_3,_ used to prepare the solutions utilized for assessing total phenol and tannin content, were purchased from Chemical Company SA, Iași, Romania. The gallic acid used to obtain the calibration curve for the total phenol content assay, the 2,2-diphenyl-1-picrylhydrazyl (DPPH) and 2,2′-azino-bis(3-ethylbenzothiazoline-6-sulfonic acid) (ABTS) reagents used for the antioxidant activity assays and hide powder used to adsorb tannins and pyrogallol used as an external standard for the tannin content assay, were all acquired from Sigma-Aldrich GmbH (Steinheim, Germany), while the Folin–Ciocâlteu reagent used for the phytochemical characterization assays was obtained from Merck KGaA (Darmstadt, Germany).

The standards used for the chromatographic assays (GC-FID and UPLC-PDA) were acquired from Sigma-Aldrich GmbH, Steinheim, Germany. For the GC-FID analysis of the extracts’ volatile compound content, nine standards were used: α- and β-pinene (99.0%), 3-carene (99.8%), α-phellandrene (98.9%), limonene (99.7%), sabinene (99.9%), myrcene (99.8%), camphene (98.9%), and tricyclene (99.8%). For the UPLC-PDA analysis of the extracts, standards from Sigma-Aldrich GmbH, Steinheim, Germany were utilized: gallic acid—GAL (99%), sinapic acid—SINAP (99%), vanillic acid—VANIL (97%), chlorogenic acid—CLR (99%), quercetin—QUER (95%), taxifolin—TAXI (85%), catechin—CAT (99%), epicatechin—EPICAT (90%), eleutheroside B—ELEB (98%), and from Extrasyntese luteolin-7-O-glucoside—LG (98%), luteolin-3′, 7-diglucoside—LDG (97%).

### 3.2. Extraction

Ultrasound assisted (UAE) and microwave-assisted extractions (MAE) of the spruce bark, were conducted in different concentration hydro-alcoholic mixtures. The extraction protocols for both extraction methods were previously described in two studies conducted on beech bark [34,65] and are briefly presented in Table 4.

Two extractions were performed with each method for every bark sample, thus resulting in two subvariants for every experimental variant. Triplicate measurements were then performed on each one of these subvariants, leading to six measurements per variant, for all of the used assays.

Eight experimental variants resulted after the extraction procedures, presented in Table 5.

### 3.3. Assessment of the Content of Total Polyphenols (TPC)

For assessing the TPC, the Folin–Ciocâlteu method was used [66], slightly modified as previously mentioned in one of our studies [61]. Briefly, 40 µL of diluted (1:5) extract were brought together in a vial with distilled water, Folin–Ciocâlteu reagent and a 20% Na_2_CO_3_ solution. This mixture was then kept at room temperature for 30 min. Finally, the absorbance was spectrophotometrically measured at a wavelength of 765 nm. For every experimental variant six measurements were performed. A calibration curve was used to express the TPC as gallic acid equivalents per gram of vegetal matrix (mg GAE/g vegetal matrix).

### 3.4. Assessment of the Content of Total Tannins (TTC)

For the TTC determination of the tested extracts, a method utilized for the determination of tannins in herbal drugs was used [67]. This method uses the Folin–Ciocâlteu assay to assess the TPC of the extracts spectrophotometrically, before and after treatment with hide powder. This procedure uses the property of tannins to get adsorbed by the hide powder, thus the difference in absorbance recorded for the tested samples before and after treatment with hide powder, indirectly represents the absorbance of the tannins adsorbed. The tannins absorbance was translated into TTC expressed as mg pyrogallol/g vegetal material by using pyrogallol as an external standard. The exact work protocol was previously described [61], but this study used a 1:50 extract dilution and a 15% Na_2_CO_3_ solution.

### 3.5. Terpenoids Content

In order to identify and quantify the potential volatile compounds present in the bark extracts, gas chromatography with flame ionization detection (GC-FID) was used. A GC with a TG-WAXMS capillary column, coupled with a FID detector was used for this procedure. The linearity interval was determined using five calibration solutions prepared in duplicates. For the calculation of the concentrations, the densities of the compounds were taken into account and a series of five dilutions was prepared from a stock solution of terpenes. The concentrations of the solutions in the calibration curves were ranged as follows: for C_α-pinene, β-pinene_ = 23.55–1177.5 mg/L, for C_camphene_ = 11.85–150.3 mg/L, for C_3-carene_ = 12.25–148 mg/L, for C_α-phellandrene_ = 0.5–10 mg/L, for C_limonene_ = 20–150.6 mg/L, for C_sabinene_ =5–50 mg/L, for C_myrcene_ = 24.27–485.4 mg/L, for C_tricyclene_ = 5.15–122.1 mg/L, respectively. If the signals given by some samples were out of linearity interval, the samples were diluted in order to fit within linearity range of the method. The precision was determined as relative standard deviation (% RSD) calculated for the three repeated measurements for the upper limit concentrations of the calibration curve (i.e., C_α-pinene, β-pinene_ = 1177.5 mg/L, C_camphene_ = 150.3 mg/L, C_3-carene_ = 148 mg/L, C_α-phellandrene_ = 10 mg/L, C_limonene_ = 150.6 mg/L, C_sabinene_ = 50 mg/L, C_myrcene_ = 485.4 mg/L, C_tricyclene_ = 122.1 mg/L).

The extracts were directly injected into the GC and helium (He) was used as a carrier gas. The identification process consisted in the comparison of the peak retention times from the standard solution chromatogram with the retention times of the peaks registered on the tested extracts chromatograms. The identified compounds were quantified based on their peak height. The detailed analysis protocol was previously described [61].

### 3.6. Individual Polyphenol Content

The individual phenolic compounds from the tested extracts were identified and quantified using ultra-performance liquid chromatography (UPLC). The detector used for the UPLC analysis consisted in a photodiode array (PDA) detector. Chromatographic separation was performed at 35 °C on a Luna C18 (2) 150 × 4.6 mm, 3 µm column (Phenomenex). The mobile phase consisted of a mixture of formic acid 0.1% (A) and acetonitrile (B). The gradient ellution was: 0–0.1 min: 90% A, 10% B; 0.1–20.1 min: 90%→ 20% A; 20.1–25.1 min: 20% A; 25.1–26.1 min: 20% A → 90% A; 26.1–30.1 min: 90% A. The flow rate was 1 mL/min and the injection volume was 20 µL. The 210 nm, 280 nm, and 370 nm wavelengths were monitored. The identification process consisted in the comparison of the retention times of the peaks on the standard solution chromatogram with those on the extracts’ chromatograms. The compounds associated with each peak were then quantified based on the peak area and identified based on the similarity of UV spectra when compared to standard solutions. This method was also described in our previous study [61].

### 3.7. Antioxidant Capacity (AC)

#### 3.7.1. DPPH Assay

The AC was assessed using the DPPH method [68]. A 50-fold dilution was applied before the assay for each experimental variant. The diluted extracts were then treated with a DPPH solution and after 30 min the absorbance was spectrophotometrically measured at 517 nm. The exact method was previously described [61]; however, in this study 150 µL of sample were used.

#### 3.7.2. ABTS Assay

In order to assess the AC by the ABTS method [69] the experimental variants were treated with the ABTS reagent and after 5–6 min the absorbance was spectrophotometrically measured at 734 nm, in a microplate reader. The exact method was also previously described [61].

### 3.8. Antibacterial Activity

The minimum inhibitory concentration (MIC) was used as an indicator of the antibacterial activity. For the MIC evaluation, the microdilution method that was previously outlined [70], was used. In addition to this method, we used resazurin to ease the visualization of bacterial growth in the wells of the microplate [71]. For the accuracy of the results, the extracts were first subjected to an evaporation step, to remove any trace of ethanol that could modify antibacterial activity.

### 3.9. Statistical Interpretation of the Data

The differences that occurred between the variants’ TPC, TTC, AC, and volatile compounds content were statistically interpreted using a one-way ANOVA test to detect potential differences in the means of the data sets. Afterward, Tukey’s post hoc test was performed if any mean difference occurred, to identify the specific variants that significantly differ (significance level α = 0.05, *p* ≤ 0.05) from each other. A Pearson correlation test was also performed to assess the linear correlation between the TPC and the AC of the extracts. These tests were performed in the GraphPad Prism 8 statistical software.

## 4. Conclusions

The results of this study indicated that differences occur between the barks collected from different locations and between the extracts obtained by different methods, regarding the phytochemical composition, antioxidant, and antibacterial activities. It has been shown that the UAE is more efficient for extracting phenolic compounds and tannins (excepting the case of the M1 variants), while the MAE generally resulted in higher yields of terpenoids (mainly α-pinene and β-pinene). Minor differences were registered between the collecting sites of the barks; however, these differences were not significant.

The antioxidant activity of the extracts attained by UAE was also higher compared to their MAE counterparts, a strong positive correlation being identified between the TPC and AC of the extracts, indicating that phenolic compounds are the main antioxidants present in the extracts and thus the primary antioxidant agents in the bark of *P. abies*.

The antibacterial effects of the spruce bark extracts were more evident against Gram-positive bacteria, all of the experimental variants having similar efficiencies against *S. aureus* (methicillin sensitive and resistant), with M1 M and MD M variants being slightly more efficient against these strains. Although, the extracts’ activity against Gram-negative bacteria was clearly weaker, the growth inhibition of *E. coli*, *K. pneumonia,* and *P. aeruginosa* still occurred for some of the experimental variants at higher MICs.

Considering the presented data, the spruce bark may represent an important source of phytochemicals with antioxidant and antibacterial activities. The valorization of this by-product and all of its bioactive constituents may lead to new natural products with important therapeutic uses. However, more data regarding the toxicological profile, doses and detailed mechanism of action are required in order to achieve this goal.

## Figures and Tables

**Table 1:** Terpenoids present in the bark of *P. abies* (mean ± st. dev. µg compound/g vegetal material).

Compound	M1 US	M1 M	M2 US	M2 M	MT US	MT M	MD US	MD M
α-pinene	1938.77 ± 3.05 ^a^	2198.33 ± 0.25 ^b^	1960.84 ± 2.97 ^c^	2005.08 ± 2.53 ^d^	1368.23 ± 3.21 ^e^	1566.11 ± 7.79 ^f^	1806.31 ± 8.09 ^g^	2014.84 ± 7.18 ^d^
β-pinene	2140.04 ± 3.59 ^a^	2997.66 ± 5.07 ^b^	3747.53 ± 22.01 ^c^	3808.67 ± 6.88 ^d^	3915.73 ± 10.04 ^e^	3936.65 ± 32.46 ^e^	4215.63 ± 6.22 ^f^	4312.24 ± 4.07 ^g^
camphene	51.11 ± 0.13 ^a^	71.60 ± 0.09 ^b^	80.77 ± 0.36 ^c^	99.74 ± 1.13 ^d^	65.96 ± 0.67 ^e^	57.17 ± 0.07 ^f^	71.12 ± 0.26 ^b^	90.56 ± 0.46 ^g^
3-carene	60.39 ± 0.14 ^a^	55.26 ± 0.09 ^b^	109.41 ± 0.23 ^c^	81.09 ± 0.03 ^d^	100.59 ± 1.06 ^e^	112.71 ± 4.08 ^c^	110.18 ± 0.15 ^c^	101.31 ± 1.80 ^e^
α-phellandrene	9.18 ± 0.17 ^a^	8.14 ± 0.08 ^b^	14.22 ± 0.16 ^c^	15.09 ± 0.03 ^d^	12.75 ± 0.34 ^e^	13.16 ± 0.08 ^e^	12.16 ± 0.09 ^f^	13.96 ± 0.12 ^c^
limonene	98.96 ± 0.14 ^a^	101.07 ± 0.06 ^b^	109.21 ± 0.05 ^c^	107.06 ± 0.08 ^d^	113.55 ± 0.11 ^e^	113.82 ± 0.30 ^e^	101.11 ± 0.03 ^b^	105.46 ± 0.47 ^f^
sabinene	22.12 ± 0.03 ^a^	23.09 ± 0.04 ^b^	26.15 ± 0.04 ^c^	26.61 ± 0.06 ^d^	67.3 ± 0.03 ^e^	67.79 ± 0.07 ^f^	75.02 ± 0.14 ^g^	81.01 ± 0.18 ^h^
myrcene	100.1 ± 0.1 ^a^	105.46 ± 0.05 ^b^	255.1 ± 0.04 ^c^	255.66 ± 0.37 ^c^	232.4 ± 4.24 ^d^	229.99 ± 0.09 ^d^	215.26 ± 0.19 ^e^	220.21 ± 0.06 ^f^
tricyclene	19.31 ± 0.17 ^a^	19.37 ± 0.11 ^a^	26.05 ± 0.06 ^b^	29.18 ± 0.06 ^c^	11.12 ± 0.06 ^d^	11.95 ± 0.08 ^e^	12.11 ± 0.07 ^e^	13.20 ± 0.07 ^f^

M1 US—*P. abies* (Maramureșului Mountains National Park site 1) extract produced by UAE, M2 US—*P. abies* (Maramureșului Mountains National Park site 2) extract produced by UAE, MT US—*P. abies* (Tașca) extract produced by UAE, MD US—*P. abies* (Dolhasca) extract produced by UAE, M1 M—*P. abies* (Maramureșului Mountains National Park site 1) extract produced by MAE, M2 M—*P. abies* (Maramureșului Mountains National Park site 2) extract produced by MAE, MT M—*P. abies* (Tașca) extract produced by MAE and MD M—*P. abies* (Dolhasca) extract produced by MAE; significant differences are represented by different letters (significance level α = 0.05).

**Table 2:** Polyphenolic compounds identified and semiquantified in the extracts (mg compound/g vegetal material).

Compound	M1 M	M1 US	M2 M	M2 US	MT M	MT US	MD M	MD US
Catechin	1.91	7.47	0.86	6.43	0.94	5.47	1.63	6.94
Epicatechin	6.72	27.09	7.41	16.86	2.02	25.59	20.93	25.71

M1 US—*P. abies* (Maramureșului Mountains National Park Site 1) extract produced by UAE, M2 US—*P. abies* (Maramureșului Mountains National Park site 2) extract produced by UAE, MT US—*P. abies* (Tașca) extract produced by UAE, MD US—*P. abies* (Dolhasca) extract produced by UAE, M1 M—*P. abies* (Maramureșului Mountains National Park site 1) extract produced by MAE, M2 M—*P. abies* (Maramureșului Mountains National Park site 2) extract produced by MAE, MT M—*P. abies* (Tașca) extract produced by MAE and MD M—*P. abies* (Dolhasca) extract produced by MAE.

**Table 3:** MICs of the tested extracts expressed as mg vegetal material/mL extract.

Bacterial Strain	MIC (mg bark/mL Extract)							
	M1 M	M1 US	M2 M	M2 US	MT M	MT US	MD M	MD US
*Staphylococcus aureus* ATCC 25923	3.125	6.25	6.25	6.25	6.25	6.25	3.125	3.125
Methicillin-resistant *Staphylococcus aureus* (MRSA) ATCC 43300	3.125	6.25	6.25	3.125	6.25	6.25	3.125	6.25
*Escherichia coli* ATCC 25922	100	50	>100	>50	100	50	100	50
*Klebsiella pneumoniae* ATCC 700603	>100	>50	>100	>50	100	>50	100	>50
*Pseudomonas aeruginosa* ATCC 27853	100	50	100	50	100	50	50	50

M1 US—*P. abies* (Maramureșului Mountains National Park site 1) extract produced by UAE, M2 US—*P. abies* (Maramureșului Mountains National Park site 2) extract produced by UAE, MT US—*P. abies* (Tașca) extract produced by UAE, MD US—*P. abies* (Dolhasca) extract produced by UAE, M1 M—*P. abies* (Maramureșului Mountains National Park site 1) extract produced by MAE, M2 M—*P. abies* (Maramureșului Mountains National Park site 2) extract produced by MAE, MT M—*P. abies* (Tașca) extract produced by MAE and MD M—*P. abies* (Dolhasca) extract produced by MAE.

**Table 4:** Extraction parameters.

Extraction Procedure	Extracted Material (Spruce Bark)	Solvent (Ethanol)	Temperature (°C)	Extraction Time (min)	Ultrasound/Microwave Power (W)	Ultrasound Frequency (kHz)
UAE	1g	70%	65	30	240	40
MAE	2g	50%	-	4	300	-

**Table 5:** Experimental variants for each collection site and extraction method used.

Collection Site	*P. abies* Bark Sample	Extraction Procedure	Extracts Attained (Experimental Variant)
Maramureșului Mountains National Park site 1 (47°47′05″ N, 24°34′20″ E)	M1	UAE	M1 US
		MAE	M1 M
Maramureșului Mountains National Park site 2 (47°47′05″ N, 24°34′20″ E)	M2	UAE	M2 US
		MAE	M2 M
Tașca (46°53′34″ N, 26°02′05″ E)	MT	UAE	MT US
		MAE	MT M
Dolhasca (47°25′49″ N, 26°36′34″ E)	MD	UAE	MD US
		MAE	MD M

## Data Availability

Data sharing not applicable.
